# Effect of *Pistacia Atlantica* Resin Oil on Anti-Oxidant, Hydroxyprolin and VEGF Changes in Experimentally-Induced Skin Burn in Rat

**DOI:** 10.29252/wjps.7.3.357

**Published:** 2018-09

**Authors:** Beydolah Shahouzehi, Gholamreza Sepehri, Sakine Sadeghiyan, Yaser Masoomi-Ardakani

**Affiliations:** 1 Student Research Committee, School of Medicine, Kerman University of Medical Sciences, Kerman, Iran;; 2 Physiology Research Center, Institute of Neuropharmacology, Kerman University of Medical Sciences, Kerman, Iran;; 3 Department of Physiology and Pharmacology, Kerman University of Medical Sciences, Kerman, Iran;; 4 Neuroscience Research Center, Kerman University of Medical Sciences, Kerman, Iran

**Keywords:** *Pistacia atlantica*, VEGF, Anti-oxidant, Burn injury, MDA

## Abstract

**BACKGROUND:**

Severe burn damage and its consequences are life threatening which can complicate patients’ health. Medicinal and traditional plants are considered as safe, natural and inexpensive source of treatment for wide variety of diseases. This study assessed beneficial effect of *Pistacia atlantica* oil on rats burn wound healing and its potential effects on malondialdehyde (MDA), vasculoendothelial growth factor (VEGF), hydroxyprolin and antioxidant status in wound area.

**METHODS:**

Thirty male rats weighing 200±10 g were randomly divided into three groups (n=10) as follows. Group 1 underwent just burn injury, Group 2 underwent burn injury and received 150 mg/kg/day *P. atlantica* oil topically, and Group 3 underwent burn injury and received 150 mg/kg/day sulfadiazine cream topically. At the end of the study (day 14), wounded areas were measured and then skin in the burn damage were dissected and anti-oxidative parameter, MDA, VEGF and hydroxyprolin were evaluated.

**RESULTS:**

*P. Atlantica* oil significantly increased antioxidant defense, VEGF, hydroxyprolin and reduced MDA levels. It could remarkably reduce wound size compared to burn control group. *P. Atlantica* oil showed more beneficial effects than sulfadiazine.

**CONCLUSION:**

*P. atlantica* resin oil could be considered as a new therapeutic agent for treatment of injuries.

## INTRODUCTION

Burn damage and its consequences lead to major problems which can complicate patients’ health. Seriously burned patients’ needs strict regular care including wound healing care and treatment, nutritional supports and control of probable infection.^1^ Pathophysiological changes in burned area caused by increased tissue temperature leads to inflammatory response and also thermal exposure can cause necrosis in burn area especially in middle parts.^2^ Increased Reactive Oxygen Species (ROSs) lead to severe damage to the cells in burn area.^3^

Wound healing is a dynamic process which consist three phases including inflammation, proliferation and maturation and cytokines and reduced local ischemia and ROSs have a pivotal role in this process. ROSs are one of the components which are participates in tissue damage. Also following thermal injury ROSs have been considered to participate in a number of pathophysiological steps. It has also been reported that ROSs are involved in burn shock and lung damage following thermal injury.^1^^,^^2^

Sepsis is the leading cause of mortality in burn units and infection is one of the major problems correlated burn injuries. Nevertheless, antibacterial remedies which used in order to eliminate infection from burn area, the infection problem still is present and need more attention.^4^ Vasculoendothelial Growth Factor (VEGF) is a multi-action growth factor that facilitates wound healing and helps tissue repair. VEGF increases inflammatory cells in damage area and also promotes migration and proliferation of endothelial cells.^4^^,^^5^

Collagen is an extracellular matrix protein which is related to wound healing and strength. Hydroxyproline is an amino acid that especially presents in collagen structure and its levels in wound parts may be as a marker of wound healing rate.^6^ Medicinal and traditional plants always considered as a good, safe and inexpensive source of remedy for many diseases. Therefore, a wide range of plants are used as pharmacological agent against diseases and improve body health.^7^^-^^10^

It has been reported that Emu oil postpones the wound healing process at inflammatory process but on the other hand Emu oil showed beneficial wound healing effect on keratinization of epidermis.^8^ Other study demonstrated that *Capparis spinosa leaves *hydro-alcoholic extract attenuates inflammation and also promoted wound healing process.^7^ One of these traditional plants that used commonly is *Pistacia atlantica* which has been reported that have many potential beneficial effects.^10^^-^^15^ *P. atlantica* is a plant which is widely distributed in Algeria, Iran, Iraq, Mediterranean and turkey.^9^

Its resin used as chewing gum and mouth freshener. *P. atlantica *resin have used as a traditional treatment for peptic ulcer disease.^11^ There are some studies about wound healing and anti-inflammatory properties of *P. atlantica* resin in animal models and also it used traditionally as a remedy for wound healing in some parts of Iran.^10^ Peksel *et al.* (2013) showed that aqueous extract of *Pistacia* leaves has radical scavenging properties.^16^ Also other studies were approved antioxidant activity of *P. atlantica.*^12^^,^^14^ It have been proved that *P. atlantica* have used for treatment of digestive diseases and also traditionally used for treatment of disorders such as colitis, gastrointestinal problems, kidney, heart and liver complications.^9^^,^^10^ Other studies reported antifungal, antiparasite and antibacterial activity of *P. atlantica.*^13^^-^^15^ In present study we evaluated ameliorative effect of *P. atlantica* resin oil on wound healing in rat which burden experimentally burn wound on skin and also we assessed *P. atlantica *resin oil effects on VEGF, hydroxyproline, and antioxidant status in wound area.

## MATERIALS AND METHODS

Thirty male Sprague-Dawley rats weighing 200±10 g were obtained from the animal care center of Kerman Neuroscience Research Center. The animals were maintained at controlled condition, 25±1 ºC and 12 h light-dark cycle and have access freely to standard chow diet and water. Our study was approved by the ethic committee of Kerman University of Medical Sciences, Kerman, Iran. *P. atlantica* resin oil which we have used in our study was prepared from Hakim-Tehrani Co. Kerman, Iran. Full compositions of *P. atlantica *oil have been reported previously.^17^^,^^18^

The animals were anesthetized by i.p. injection of Ketamin and Xylazine (60 and 4 mg/kg, respectively). The dorsal side of the animals were shaved and then induction of burn damage conducted by an aluminum plaque (2.5x2.5 cm) on the shaved skin of rats for 15-20 seconds which was heated to 100ºC to create a deep dermal burn wound.^19^^,^^20^ Duration of study was 14 days after burn injury and animals were divided into three groups (n=10) randomly as follow: Group 1 underwent just burn injury, Group 2 underwent burn injury and received 200 mg/kg/day of *P. atlantica* resin oil topically, and Group 3 underwent burn injury and received 200 mg/kg/day of sulfadiazine cream topically.

At the end of the study animals were anesthetized and sacrificed then burned skin were incised and separated. Samples were homogenized by Ultrasonic Processor (Hielscher, UP200H) in cold phosphate buffered saline (PBS, pH=7.4) and then centrifuge at 4°C and 15000 rpm for 15 min. Supernatants were separated and aliquoted, then maintained at -80 until further experiments. Superoxide dismutase (SOD), Glutathione peroxidase (GPX), Total Antioxidant Status (TAS), Malondialdehyde (MDA), VEGF and hydroxyproline were measured in supernatant.

The wound contractions was reported as percent and were calculates by following formula:^21^ %wound contraction=(wound area [day 1]-wound area [day 14]/wound area [day 1]×100

Measurement of VEGF, hydroxyprolin, MDA, TAS, SOD and GPX were conducted by specific kits (Hydroxyproline Elisa assay kit E0511Ra, Crystal Ray Biotech inc.; VEGF Elisa assay kit E0659Ra, Crystal Ray Biotech inc.; SOD, GPX and TAS conducted by using specific kits supplied from RANDOX laboratories Ltd. (*TAS*, Cat. No. NX2332; *SOD*, Cat. No. SD125; and *GPX*, Cat. No. RS505). MDA was measured as thiobarbituric acid-reactive substances (TBARS) at 534 nm and 1,1,3,3-tetramethoxypropane was used to plot calibration curve. The data were expressed as mean±SEM. For comparison between groups One-way ANOVA test followed by post hoc Tukey’s performed to compare mean differences between groups, and *p*<0.05 was considered as statistically significant.

## RESULTS

**Fig. 1:**
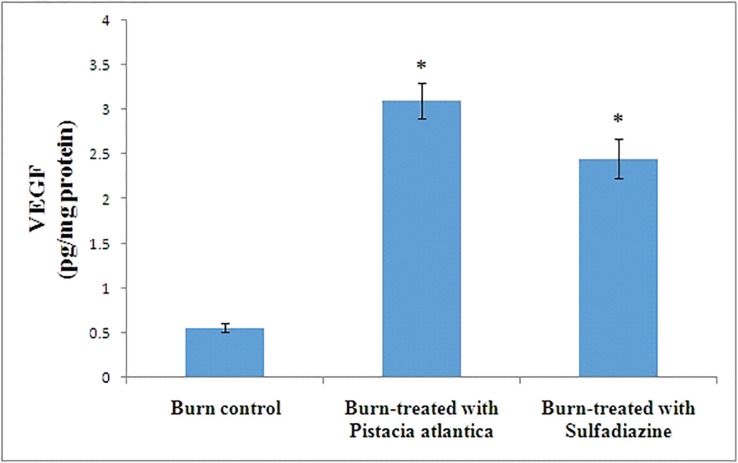
VEGF levels in burn wound area. Group 1, burn control; group 2, burn-treated with *P. atlantica* resin oil topically; group 3, burn-treated with sulfadiazine. *statistically significant compared to Burn control group, #statistically significant compared to burn-treated with sulfadiazine group

**Fig. 2:**
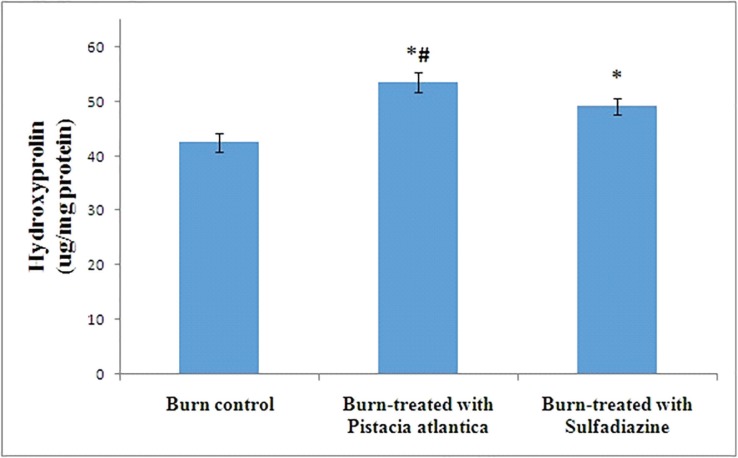
Hydroxyproline levels in burn wound area. Group 1, burn control; group 2, burn-treated with *P. atlantica* resin oil topically; group 3, burn-treated with sulfadiazine. *statistically significant compared to Burn control group, # statistically significant compared to burn-treated with sulfadiazine group

Our results showed that *P. atlantica* resin oil significantly increase antioxidant defense, VEGF, hydroxyprolin and reduced MDA levels (Table 1, Figures 1 and 2). Also *P. atlantica* compared to sulfadiazine significantly increased SOD, GPX, TAS and hydroxyproline (Table 1, Figures 1 and 2). *P. atlantica* remarkably reduced wound size compared to burn control group (Table1)

**Table 1:** Wound size, MDA and anti-oxidative parameters

**Burn**	**Wound contraction (%)**	**MDA (nmol/mg protein)**	**SOD (U/mg protein)**	**GPX (U/mg protein)**	**TAS (mmol/mg protein)**
Burn non-treated control	71.2±3.4	6.1±0.4	2.0±0.35	7.9±0.07	0.62±0.04
Burn-treated with *P. atlantica*	98.6±2.5*	1.5±0.14*	6.0±0.43*#	25.1±1.2*#	1.93±0.1*#
Burn-treated with sulfadiazine	94.7±4.1*	2.2±0.22*	4.7±0.35*	19.0±1.18*	1.52±0.07*

* Significant compared to control group,

# significant compared to sulfadiazine treated group, (n=10), *p*<0.05 considered as statistically significant

## DISCUSSION

Our results showed that *Pistacia atlantica* resin oil has remarkable antioxidant properties in experimentally induced- burn wound. Also we found that its resin oil is capable of increase the VEGF and hydroxyproline levels in wound area. Bozorgi *et al.* have reported that resin of *P. atlantica *has been used as treatment for some diseases and complicated condition such as digestive, hepatic, and kidney diseases. Also it demonstrated that the gum resin of *P. atlantica *can be used for wound healing and treatment of gastrointestinal problems.^9^

Previous studies have showed that *P. atlantica *resin oil have many compounds and α-pinene (about 45-70%) is the major components and it seems that α-pinene is the effective substance present in resin oil.^9,17,22,23^ Also, Memariani *et al*. (2017) demonstrated that α-pinene is the main component of *P. atlantica* oil and 2g/kg of *P. atlantica* oil was not harmful or toxic in vivo. They reported that *P. atlantica* oil showed protective effect against experimentally ethanol induced gastric ulcer.^24^ Also it has been reported that α-Pinene showed significant low toxicity.^18^

*P. atlantica* oil antioxidative properties have been reported previously.^12^^,^^16^^,^^25^ Rezaie *et al*. have reported that *P. atlantica* oil has remarkable antioxidant activities compared with positive control.^14^ Koizumi *et al*. showed that severe burn damage cause promotion of free radicals which in turn result in vasodilatation and SOD can act as a protective factor against vasodilatation.^26^ Here we proved that *P. atlantica* resin oil significantly compared with burn control group elevated SOD levels. 

We also found that *P. atlantica* resin oil is a potent antioxidant ointment which significantly improved antioxidant status of wound area in rats. Also we found that *P. atlantica* resin oil is rather potent than sulfadiazine to fight against free radicals present in burn wound. Farahpour *et al.* showed that *P. atlantica* hydroalcoholic hull extract showed antioxidant activity even higher than ascorbic acid, their finding are consistent with our antioxidative data about *P. atlantica* resin oil.^22^

It showed that *P. atlantica* resin oil has important antimicrobial activity against bacteria which showed resistance against some common antimicrobial drugs.^15^ Also other studies reported antimicrobial activity of *P. atlantica* oil.^14^^,^^25^ Therefore, *P. atlantica* resin oil in addition to increase antioxidants also has antimicrobial properties and it reduce the chance of infection and accelerate the process of wound healing. 

Galiano *et al.* demonstrated that topical VEGF increased growth factor in damaged area and also recruits bone marrow derived cells which have remarkable role in wound repair. All together they showed that topical application of VEGF has wound healing properties.^5^ Haghdoost *et al.* showed that *P. atlantica* increases bFGF and PDGF and therefore result in angiogenesis.^20^ We showed that *P. atlantica* resin oil promotes VEGF levels in wound area in burn model injury in rats and this could explains its beneficial effects on wound contraction and repair in this study. Therefore, Galiano *et al.* and Haghdoost and colleagues reports are confirming our data regarding VEGF variations in wound area.^5^^,^^20^

Collagen is an extracellular matrix protein which is related to wound contraction and strength. Collagen turnover directly related to free hydroxyproline and quantification of hydroxyproline can be considered as a good parameter to monitor collagen turnover.^22^^,^^27^ Hamidi and colleagues showed that *P. atlantica* oil changed collagen pattern and caused organized collagen fiber after three weeks.^28^ Farahpour *et al.* showed that *P. atlantica* hydroalcoholic hull extract as ointment increased hydroxyproline content and histological study showed that collagen score increased significantly. Also they found that *P. atlantica* hydroalcoholic hull extract promotes fibroblasts proliferation and therefore decline inflammation.^22^

IIango *et al.* evaluated methanol extract of L. acidissima on SOD, catalase, hydroxyproline, and epithelialization. They found that methanol extract of L. acidissima increase antioxidant SOD and also promotes hydroxyproline and epithelialization, therefore showed significant dose dependent wound healing activity.^27^ We showed that *P. atlantica* resin oil increased hydroxyproline levels in wound area and this could be considered as a collagen turnover marker. Therefore, *P. atlantica* resin oil wound healing and contraction activity can be related to elevation of collagen turnover and consequently hydroxyproline. 

Epithelialization is considered as an important factor which serves as a defining parameter of affluent wound contraction.^28^ Mehrabani *et al.* used *P. atlantica* oil in combination with three other oils from sesame (*Sesamum indicum *L.), hemp (*Cannabis sativa *L.) and walnut (*Juglans regia *L.) as a combined formula. This new formula increased wound contraction and accelerated epithelialization.^21^ Hamidi *et al*. showed that *P. atlantica* oil as gels ameliorated epithelialization which is as a marker of wound contraction. Also they showed that topical *P. atlantica* oil in rat with experimental wound cause biochemical and morphological promotion compared with control group.^28^

Therefore, according to these reports about *P. atlantica* oil we can anticipate that increased epithelialization and other beneficial histological changes can be considered as potential mechanism in addition to elevation of VEF and hydroxyproline and antioxidant status to increase. Finally according to previous studies *P. atlantica* resin oil has many beneficial properties and low toxicity, and our finding showed that Pistacia atlantica resin oil has antioxidant effects and increases VEGF and hydroxyproline, therefore, it seems that *P. atlantica* resin oil is a good, safe and potent candidate as a new therapeutic topical ointment for wound healing.
